# Increased serum salusin-α by aerobic exercise training correlates with improvements in arterial stiffness in middle-aged and older adults

**DOI:** 10.18632/aging.102678

**Published:** 2020-01-09

**Authors:** Shumpei Fujie, Natsuki Hasegawa, Kiyoshi Sanada, Takafumi Hamaoka, Seiji Maeda, Jaume Padilla, Luis A. Martinez-Lemus, Motoyuki Iemitsu

**Affiliations:** 1 Faculty of Sport and Health Sciences, University of Tsukuba, Ibaraki, Japan; 2 Research Fellow of Japan Society for the Promotion of Science, Tokyo, Japan; 3 Dalton Cardiovascular Research Center, University of Missouri, Columbia, MO 65211, USA; 4 Research Organization of Science and Technology, Ritsumeikan University, Shiga, Japan; 5 Faculty of Sport and Health Science, Ritsumeikan University, Shiga, Japan; 6 Sports Medicine for Health Promotion, Tokyo Medical University, Tokyo, Japan; 7 Department of Nutrition and Exercise Physiology, University of Missouri, Columbia, MO 65201, USA; 8 Department of Medical Pharmacology and Physiology, University of Missouri, Columbia, MO 65212, USA

**Keywords:** aging, exercise training, pulse wave velocity, cardiovascular disease risk factors, salusin-α

## Abstract

Aging causes arterial stiffening which can be mitigated by increased physical activity. Although low circulating levels of salusin-α are associated with cardiovascular disease, whether salusin-α decreases with aging and whether the reduced arterial stiffening occurring with exercise training is associated with increased serum salusin-α is unknown. Herein we assessed carotid-femoral pulse wave velocity (cfPWV), systolic (SBP) and diastolic (DBP) blood pressures in a cross-sectional study that compared young (20-39-year-old, n=45) versus middle-aged and older (40-80-year-old, n=60) subjects. We also performed an interventional study in which 36 young and 40 middle-aged and older subjects underwent eight weeks of aerobic exercise training. In the cross-sectional study, serum salusin-α levels were lesser in middle-aged and older subjects compared to young individuals and negatively correlated with age, SBP, DBP, or cfPWV. In the interventional study, exercise training increased serum salusin-α in middle-aged and older subjects. Notably, negative correlations were noted between the exercise training-induced changes in serum salusin-α and cfPWV, SBP and DBP. Results indicate that advanced age associates with low circulating salusin-α, the levels of which can be augmented by exercise training. Importantly, increased serum salusin-α with exercise correlates with improvements in arterial stiffness and a reduction in blood pressure.

## INTRODUCTION

Aging is associated with an increased prevalence of cardiovascular disease (CVD) risk factors, among which is arterial stiffening [1–3]. Notably, aortic pulse wave velocity (PWV), i.e., the in vivo gold-standard measurement of aortic stiffness, increases 6–8% with each decade of life up to 50 years and by 18% thereafter [4, 5]. This progressive stiffening of the vasculature augments the risk for coronary artery disease, heart failure, atherosclerosis and stroke [1–3]. In comparison, reduced arterial stiffness is associated with a decreased risk for such life-threatening CVD and events [6, 7]. Consequently, much effort has been placed on deciphering the mechanisms that control arterial stiffness, and on developing interventions to reduce arterial stiffness and its associated cardiovascular consequences [8]. Among such interventions is aerobic exercise training (AT), which is known to reduce arterial stiffness, blood pressure and overall CVD risk in old adults [9, 10]. Indeed, AT is considered a first-line therapeutic strategy for reducing the incidence of CVD in the elderly [11–13].

Although the mechanisms by which AT reduces arterial stiffness and other CVD risk factors have not been completely elucidated, the anti-inflammatory effects of AT are thought to contribute to improvements in vascular function and compliance. This is supported by the synergistic, yet independent, cross-talk that exists between inflammation, atherosclerosis and arterial aging [14]. Indeed, in the elderly, arteriosclerosis is deemed the consequence of chronic vascular inflammation with associated endothelial dysfunction and thickening of the arterial wall [15]. Therefore, it is likely that the lessened chronic vascular inflammation associated with AT is partially responsible for the ameliorating effects of AT on aging-associated arterial stiffening [16–18]. In this regard, salusin-α is an endogenous bioactive peptide consisting of 28 amino acid residues that attenuates inflammatory responses in vascular cells [19, 20]. Although expressed and synthesized ubiquitously within human tissues, including the central nervous system, vascular smooth muscle cells, endothelial cells and the kidney [19], salusin-α has been particularly shown to reduce the cytotoxic effects of tumor necrosis factor-α (TNF-α) in cultured human umbilical vein endothelial cells [20]. Evidence also suggests that salusin-α may be associated with mechanisms that reduce arterial stiffness, as circulating levels of the peptide are negatively correlated with brachial-ankle PWV and arterial wall thickness [21]. A role for salusin-α on vascular health is further supported by evidence indicating that patients with coronary artery disease and hypertension have lower circulating salusin-α compared to healthy subjects [21–25]. Importantly, salusin-α may have both chronic and acute effects on the cardiovascular system, as rats treated with this bioactive peptide exhibit acute dose-dependent reductions in blood pressure [18]. In addition, the expression of salusin-α is modulated by angiotensin II via the activation of Janus kinase (Jak)-2, which is a well-known regulator of blood pressure [26, 27]. Herein, we hypothesized that circulating salusin-α levels decrease with age and that this occurs concomitantly with the development of other CVD risks factors including arterial stiffening. We further hypothesized that, in middle-aged and older adults, AT is associated with increased circulating salusin-α levels, while decreasing arterial stiffness and other CVD risk factors.

## RESULTS

### Cross-sectional study

**Figure 1:**
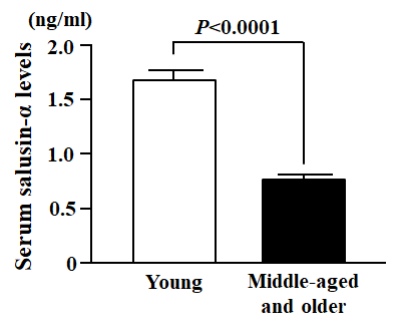
**Comparison of serum salusin-α levels between Young and Middle-aged and older subjects.** Data are expressed as means ± SE.

In the cross-sectional study, body mass index (BMI), carotid-femoral PWV (cfPWV), systolic blood pressure (SBP), diastolic blood pressure (DBP), mean blood pressure (MBP), common carotid intima-media thickness (ccIMT) and the levels of total cholesterol and triglycerides were significantly greater in the Middle-aged and older group than in the Young group (Table 1). In contrast, height and serum salusin-α levels in the middle-aged and older group were significantly lesser than in the young group (Table 1 and Figure 1).

**Table 1:** Comparison of characteristics between the Young and Middle-aged and older groups.

	**Young**	**Middle-aged and older**
Subjects (Male/Female), n	45 (25/20)	60 (24/36)
Age, years	21.4 ±0.5	67.4±0.8 *
Height, cm	167.7 ± 1.7	159.5 ±1.2 *
Body weight, kg	60.6 ±1.5	58.1 ±1.4
BMI, kg/m^2^	21.4 ± 0.3	22.8±0.5 *
Total cholesterol, mg/dl	175.7 ±5.0	222.6 ±4.5 *
HDL cholesterol, mg/dl	65.6 ±1.9	72.9 ±3.0
Triglycerides, mg/dl	69.4 ±5.1	112.5 ±10.5 *
Salusin-α levels, ng/ml	1.68 ± 0.09	0.76 ±0.05 *
HR, bpm	58.4 ±1.4	59.6 ±1.1
SBP, mmHg	112.9 ±1.9	130.3 ±2.1 *
DBP, mmHg	63.6 ±1.3	76.9 ±1.3 *
MBP, mmHg	80.0 ±1.5	94.7 ±1.5 *
cfPWV, cm/s	790.8±24.3	1134.1 ±35.0 *
ccIMT, mm	0.521 ±0.009	0.750±0.015 *

BMI: body mass index, HDL: high-density lipoprotein, HR: heart rate, bpm: beats per minute, SBP: systolic blood pressure, DBF: diastolic blood pressure, MBP: mean blood pressure, cfPWV: carotid-femoral pulse wave velocity, ccIMT: common carotid intima-media thickness.

Values are means and SE. * P<0. 05 vs. Young

**Figure 2:**
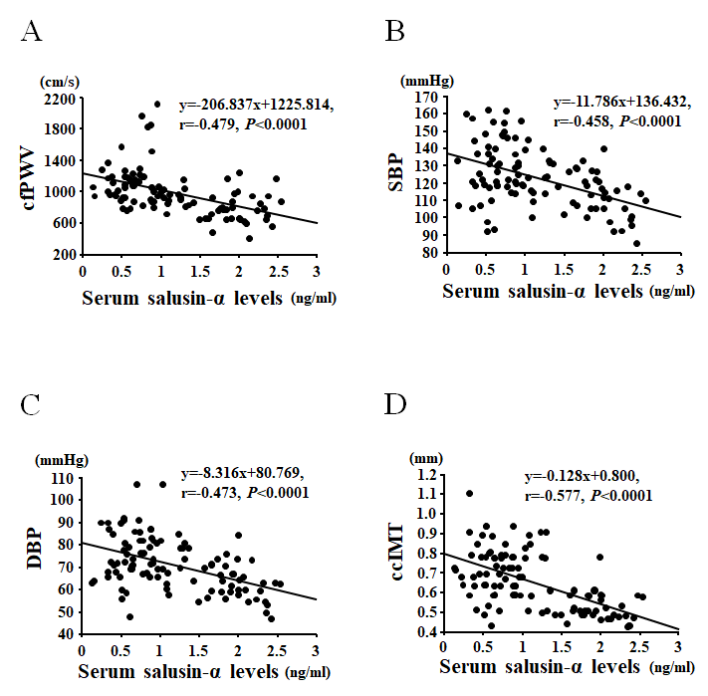
Correlations between serum salusin-α levels and carotid-femoral pulse wave velocity (cfPWV; **A**), systolic blood pressure (SBP; **B**), diastolic blood pressure (DBP; **C**), or common carotid intima-media thickness (ccIMT; **D**).

Serum salusin-α levels were negatively correlated with 1) age (r = -0.698, *P* < 0.0001); 2) total cholesterol (r = -0.374, *P* < 0.0001); 3) triglycerides (r = -0.336, *P* < 0.001); 4) SBP (r = -0.458, *P* < 0.0001); 5) DBP (r = -0.473, *P* < 0.0001); 6) MBP (r = -0.486, *P* < 0.0001); 7) cfPWV (r = -0.479, *P* < 0.0001); 8) ccIMT (r = -0.577, *P* < 0.0001) (Table 2 and Figure 2). In the multiple regression analysis using serum salusin-α levels as the dependent variable, MBP (*β* = -0.277, *P* < 0.05) and cfPWV (*β* = -0.299, *P* < 0.05) were independent factors associated with serum salusin-α levels. However, BMI and triglycerides were not independently associated with serum salusin-α levels.

**Table 2:** Correlations between serum salusin-α levels and other variables.

	**Serum salusin-α levels, ng/ml**	
	**r**	**P**
Age, years	-0.698	<0.0001
Height, cm	0.178	0.0701
Body weight, kg	-0.007	0.9441
BMI, kg/m^2^	-0.186	0.0581
Total cholesterol, mg/dl	-0.374	<0.0001
HDL cholesterol, mg/dl	-0.015	0.8780
Triglycerides, mg/dl	-0.336	0.0005
HR, bpm	-0.047	0.6358
SBP mmHg	-0.458	<0.0001
DBP, mmHg	-0.473	<0.0001
MBP, mmHg	-0.486	<0.0001
cfPWV, cm/s	-0.479	<0.0001
ccIMT, mm	-0.577	<0.0001

BMI: body mass index, HDL: high-density lipoprotein, HR: heart rate, bpm: beats per minute, SBP: systolic blood pressure, DBP: diastolic blood pressure, MBP: mean blood pressure, cfPWV: carotid-femoral pulse wave velocity, ccIMT: common carotid intima-media thickness.

Age was positively correlated with cfPWV (r = 0.636, P < 0.0001), SBP (r = 0.523, *P* < 0.0001), DBP (r = 0.545, *P* < 0.0001), MBP (r = 0.558, *P* < 0.0001) and ccIMT (r = 0.787, *P* < 0.0001).

### Interventional study

**Figure 3:**
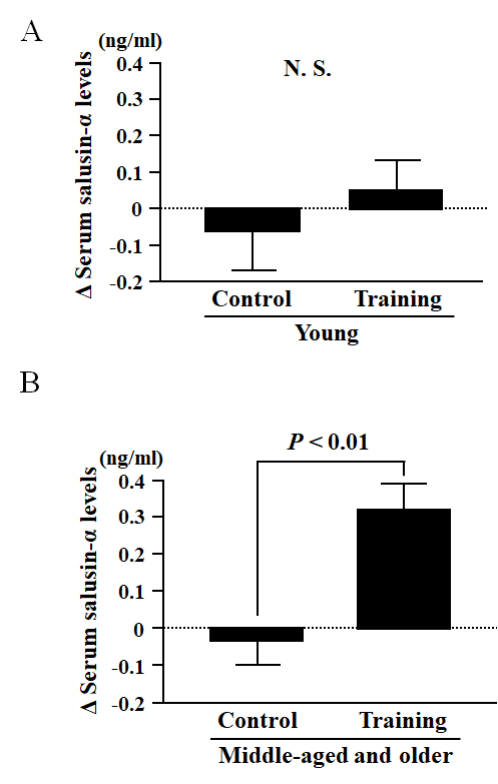
Comparison of the change in serum salusin-α levels before and after an eight-week intervention of aerobic exercise training (Training group) or sedentarism (Control group) in Young (**A**) and Middle-aged and older (**B**) subjects. Data are expressed as means ± SE.

In the interventional study, no significant differences were found in any variable measured for comparisons between the Young-AT and Young-Con groups at baseline (Table 3). However, when comparing the changes that occurred in response to AT versus control, changes in peak oxygen uptake (${\dot{\text{V}}\text{O}}_{2\text{peak}}$) were significantly greater in the Young-AT than in the Young-Con group (Table 4). In contrast, changes in body weight, heart rate (HR) and SBP were significantly lesser in the Young-AT than the Young-Con group (Table 4). There were no significant differences in BMI, total cholesterol, high-density lipoprotein (HDL) cholesterol, triglycerides, daily physical activity, DBP, cfPWV, ccIMT or serum salusin-α levels between the Young-AT and Young-Con groups (Table 4 and Figure 3A). Furthermore, the changes in salusin-α levels induced by AT were not correlated with any other variables measured in the Young-AT group (Table 5).

**Table 3:** Comparison of characteristics at baseline between Control and Training groups in the Young or Middle-aged and older groups.

	**Young-Pre**		**P value**	**Middle-aged and older-Pre**		**P value**
	**Control**	**Training**		**Control**	**Training**	
Subjects (Male/Female), n	9 (5/4)	27 (15/12)		14 (6/8)	26 (10/16)	
Age, years	21.4±0.2	21.3±0.8	0.912	67.5±1.7	66.8±1.4	0.756
Height, cm	167.7±4.4	167.5±1.9	0.967	161.0±2.5	159.2±1.9	0.574
Body weight, kg	61.7+3.7	59.8±1.8	0.616	56.3±3.5	60.2±2.0	0.311
BMI, kg/m^2^	21.8±0.7	21.1±0.3	0.351	21.6±1.1	23.9±0.7	0.068
Total cholesterol, mg/dl	166.9±6.7	183.7±7.4	0.222	225.4±9.2	217.6±6.1	0.467
HDL cholesterol, mg/dl	67.4±3.6	66.3±2.8	0.835	74.9±6.1	65.0±3.5	0.140
Triglycerides, mg/dl	65.2+8.8	73.7±7.9	0.567	114.1±27.0	124.5±15.6	0.724
Salusin-α levels, ng/ml	1.80±0.12	1.63±0.13	0.468	0.80±0.07	0.66±0.08	0.261
HR, bpm	56.4±2.7	59.5±2.0	0.566	59.4±2.2	60.3±1.6	0.740
SBP, mmHg	111.3±4.2	113.2±2.7	0.720	133.2±4.0	129.0±3.4	0.444
DBP, mmHg	62.1±2.2	64.7±1.9	0.473	77.4±2.3	77.2±2.4	0.965
MBP, mmHg	78.6±2.8	80.9±2.1	0.687	96.1±2.7	94.5±2.5	0.698
cfPWV, cm/s	720.4±38.8	809.3±23.2	0.169	1101.7±85.0	1119.6±30.2	0.811
ccIMT, mm	0.526±0.020	0.518±0.011	0.751	0.738±0.030	0.770±0.023	0.417
VO_2peak_, ml/kg/min	40.4±2.0	43.5±1.4	0.257	27.0±1.8	24.1±0.9	0.122
Physical activity, kcal/day	2355.2±155.7	2563.6±134.6	0.776	1763.0±180.7	1986.4±59.6	0.312

BMI: body mass index, HDL: high-density lipoprotein, HR: heart rate, bpm: beats per minute, SBP: systolic blood pressure, DBP: diastolic blood pressure, MBP: mean blood pressure, cfPWV: carotid-femoral pulse wave velocity, ccIMT: common carotid intima-media thickness, VO_2peak_: peak oxygen uptake. Values are means and SE.

**Table 4:** Comparison of changes before and after the 8-week exercise training between Control and Training groups in the Young or Middle-aged and older groups.

	**Young**		**P value**	**Middle-aged and older**		**P value**
	**Control**	**Training**		**Control**	**Training**	
Δ BMI, kg/m^2^	0.02±0.16	-0.24±0.10	0.195	0.14±0.10	-0.29±0.13	0.033
Δ Total cholesterol, mg/dl	-6.44±7.41	-11.19±3.40	0.517	-5.36±4.19	-2.31±4.33	0.650
Δ HDL cholesterol, mg/dl	-5.89±1.60	-0.59±1.54	0.070	-1.00±2.73	-0.42±1.70	0.852
Δ Triglycerides, mg/dl	-4.33±6.97	3.78±5.62	0.448	1.93±7.91	-13.27±10.52	0.334
Δ Salusin-α levels, ng/ml	-0.07±0.11	0.06±0.08	0.427	-0.04±0.06	0.33±0.07	0.001
Δ HR, bpm	0.56±0.90	-5.27±0.88	0.009	-2.43±0.97	-2.62±1.63	0.937
Δ SBP, mmHg	2.00±2.65	-3.91±1.25	0.032	-2.21±1.81	-10.10±2.00	0.013
Δ DBP, mmHg	-0.39±2.20	-2.50±1.20	0.391	-0.86±1.34	-4.64±1.12	0.044
Δ MBP, mmHg	0.40±2.25	-2.97±1.14	0.163	-1.31±1.41	-6.44±1.26	0.062
Δ cfPWV, cm/s	36.4±61.2	-49.7±23.8	0.122	-21.2±19.8	-138.4±19.8	0.015
Δ ccIMT, mm	0.001±0.001	0.001±0.001	0.200	0.001±0.001	-0.003±0.002	0.298
ΔVO_2peak_, ml/kg/min	0.12±1.15	3.78±0.72	0.014	-0.29±0.48	4.63±0.48	<0.001
Δ Physical activity, kcal/day	-10.6±36.4	-97.5±69.1	0.317	-31.9±45.4	-32.5±21.1	0.992

A: change in value before and after the 8-week exercise training, BMI: body mass index, HDL: high-density lipoprotein, HR: heart rate, bpm: beats per minute, SBP: systolic blood pressure, DBP: diastolic blood pressure, MBP: mean blood pressure, cfPWV: carotid-femoral pulse wave velocity, ccIMT: common carotid intima-media thickness, VO_2peak_: peak oxygen uptake. Values are means and SE.

**Table 5:** Correlations between changes in serum salusin-α levels and other variables in Training groups.

	**Young**		**Middle-aged and older**	
	**Δ Serum salusin-α levels, ng/ml**			
	**r**	**P**	**r**	**P**
Δ BMI, kg/m^2^	-0.063	0.7530	-0.066	0.7489
Δ Total cholesterol, mg/dl	0.272	0.1704	0.237	0.2433
Δ HDL cholesterol, mg/dl	0.289	0.1432	0.205	0.3150
Δ Triglycerides, mg/dl	0.006	0.9761	-0.348	0.0815
Δ HR, bpm	-0.244	0.2297	-0.246	0.2266
Δ SBP, mmHg	0.046	0.8206	-0.591	0.0015
Δ DBP, mmHg	0.337	0.0855	-0.442	0.0237
Δ MBP, mmHg	0.253	0.2025	-0.570	0.0023
Δ cfPWV, cm/s	-0.166	0.4269	-0.597	0.0013
Δ ccIMT, mm	0.183	0.3600	-0.484	0.0122

Δ: change in value before and after the 8-week exercise training, BMI: body mass index, HDL: high-density lipoprotein, HR: heart rate, bpm: beats per minute, SBP: systolic blood pressure, DBP: diastolic blood pressure, MBP: mean blood pressure, cfPWV: carotid-femoral pulse wave velocity, ccIMT: common carotid intima-media thickness.

**Figure 4:**
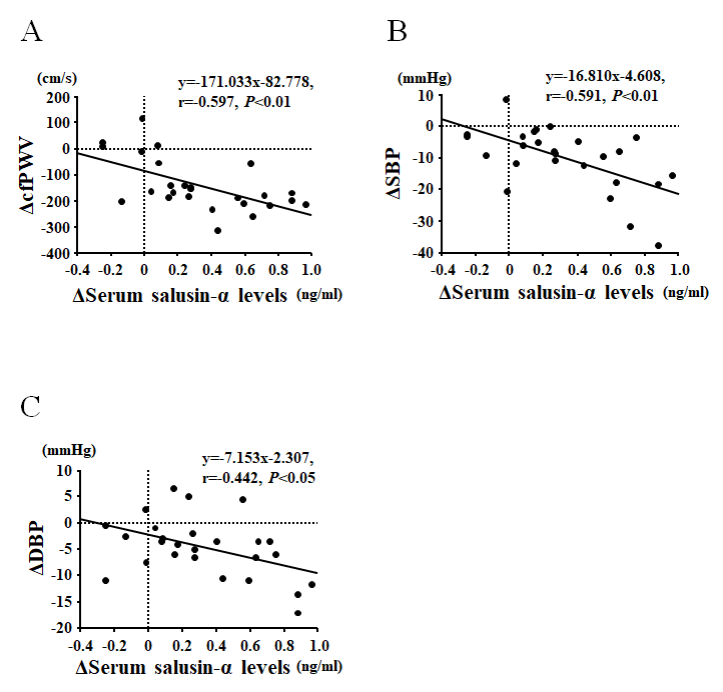
Correlations between the change in serum salusin-α levels and carotid-femoral pulse wave velocity (cfPWV; **A**), systolic blood pressure (SBP; **B**), and diastolic blood pressure (DBP; **C**) before and after an eight-week aerobic exercise training intervention in Middle-aged and older subjects.

In the Middle-aged and older group, there were no significant differences in any of the parameters measured between the AT and Con groups at baseline (Table 3). However, changes before and after the intervention in ${\dot{\text{V}}\text{O}}_{2\text{peak}}$ and serum salusin-α levels were significantly greater in the Middle-aged and older-AT group than in the Middle-aged and older-Con group (Table 4 and Figure 3B). In contrast, changes in BMI, SBP, DBP, MBP and cfPWV were significantly lesser in the Middle-aged and older-AT group than the Middle-aged and older-Con group (Table 4). There were no significant differences in changes in total cholesterol, HDL cholesterol, triglycerides, daily physical activity, HR and ccIMT between the Middle-aged and older-AT and Middle-aged and older-Con groups (Table 4). Furthermore, AT-induced changes in serum salusin-α levels were negatively correlated with AT-induced changes in cfPWV (unadjusted: *r* = -0.597, *P* < 0.01, Figure 4A; Table 5; adjusted for age: *β* = -0.682, *P* < 0.001), SBP (unadjusted: *r* = -0.591, *P* < 0.01, Figure 4B; Table 5; adjusted for age: *β* = -0.593, *P* < 0.01), DBP (unadjusted: *r* = -0.442, *P* < 0.05, Figure 4C; Table 5; adjusted for age: *β* = -0.498, *P* < 0.05) and MBP (unadjusted: *r* = -0.570, *P* < 0.01, Table 5; adjusted for age: *β* = -0.585, *P* < 0.01) but were not correlated with AT-induced changes in any other parameter measured in Middle-aged and older-AT group (Table 5).

## DISCUSSION

Herein, we report that arterial stiffening and other CVD risk factors associated with aging are negatively correlated with serum levels of the anti-inflammatory bioactive peptide, salusin-α. We also show that, in middle-aged and older adults, AT increases circulating salusin-α levels and these effects occur in parallel with an amelioration of arterial stiffening and additional CVD risk factors. Specifically, these main findings are supported by data from the cross-sectional study demonstrating that serum salusin-α levels are negatively correlated with age, arterial stiffness (cfPWV), SBP, DBP, ccIMT, and serum levels of total cholesterol and triglycerides. This indicates that salusin-α levels diminish with age and that this reduction associates with the appearance of multiple CVD risk factors. Notably, data from the interventional study demonstrate that AT-induced changes in serum salusin-α are correlated with changes in cfPWV, SBP and DBP. To the best of our knowledge, this is the first evidence relating low salusin-α levels in circulation with advanced age and demonstrating that AT increases circulating salusin-α, while concurrently decreasing arterial stiffness and blood pressure in middle-aged and older adults.

Aging is associated with a stiffening of the arteries that precedes the development of clinical hypertension and other CVD risk factors. During the arterial stiffening process there is an increased degradation of elastin structures within the vascular wall and an augmented production, deposition and cross-linking of collagen [28–30]. It is also shown that the vascular wall becomes thicker and that the smooth muscle cells within the wall increase their fibrillar actin stress fibers and become stiffer as well [30–32]. As these processes of arterial stiffening are particularly prevalent in conduit arteries, the end result is an increase in PWV that carries pulse pressure waves to the microcirculation and eventually cause end-organ damage. Therefore, a better understanding of the processes controlling arterial stiffening and associated CVD risk factors with aging is of paramount importance.

Although the mechanism by which blood vessels become stiffer with age has not been completely elucidated, multiple lines of evidence indicate that concomitant with arterial stiffening there is an increase in inflammatory signals present in the circulation and the vascular wall [4, 15]. Therefore, much work has been devoted to understanding the mechanisms that modulate vascular inflammation during the aging process. In this regard, previous studies showed that salusin-α attenuates endothelial cell inflammation in response to cytokines such as TNF-α [20]. In addition, evidence exists indicating that circulating salusin-α levels negatively correlate with arterial stiffness [25]. Therefore, it is plausible that the decrease in serum salusin-α that we observe with aging may contribute to arterial stiffening by favoring a pro-inflammatory vascular phenotype. It is also widely known that increased arterial stiffness leads to high blood pressure [33]. In contrast, administration of salusin-α dose-dependently decreases mean arterial pressure [19]. Moreover, previous studies showed that circulating salusin-α levels are negatively related to PWV or IMT in patients with essential hypertension [21, 25]. In our cross-sectional study, negative correlations were observed between serum salusin-α and cfPWV, blood pressure, and ccIMT in healthy adults across different ages. Therefore, low levels of salusin-α in circulation may have predictive value for CVD risk not only in hypertensive patients but also in healthy middle-aged and older adults. In addition, although the mechanisms by which salusin-α decreases blood pressure are not completely known, it is reported that Janus kinase (Jak)-2 inhibits salusin-α. Jak-2 is a well know regulator of blood pressure that is upregulated by angiotensin II [26, 27] and angiotensin II is also a primary regulator of blood pressure and an instigator of arterial stiffening [34–36]. Thus, it is plausible that salusin-α counteracts the pressor and arterial stiffening effects of angiotensin II.

AT is a well-known intervention that decreases arterial stiffness and other CVD risk factors. In our interventional study, AT decreased arterial stiffness and increased serum salusin-α levels in middle-aged and older subjects, but not in young subjects. We also observed that the increase in serum salusin-α was accompanied by a decrease in blood pressure. Thus, it is possible that the reduced arterial stiffness observed with AT can be attributed to the anti-inflammatory effect of salusin-α in cells of the vascular wall. It could also be speculated that AT-associated reductions in circulating angiotensin-II favor the production of salusin-α and its anti-inflammatory effects. All this is further supported by reports indicating that circulating levels of salusin-α are lesser in patients with hypertension [21, 23–25] than in healthy controls, and by data showing that AT decreases arterial stiffness in similar patient populations [37]. Whether increasing circulating salusin-α levels can causatively reduce arterial stiffness and improve vascular function in the setting of CVD risk factors, including hypertension, hyperlipidemia or hyperglycemia remains to be determined. Nonetheless, it is conceivable that increased salusin-α levels might represent a mechanism by which AT reduces arterial stiffness in middle-aged and older adults. Furthermore, increased serum salusin-α levels might also serve as a biomarker of low CVD risk.

In conclusion, we found that eight weeks of AT reversed aging-induced decreases in circulating salusin-α levels, while concurrently reducing arterial stiffness and other CVD risk factors in healthy middle-aged and older adults. As the changes in circulating salusin-α levels induced by AT were negatively correlated with changes in arterial stiffness and CVD risk, our results support the notion that increased circulating levels of salusin-α by AT may contribute to a reduction in arterial stiffness and blood pressure in middle-aged and older adults.

## MATERIALS AND METHODS

### Subjects: Cross-sectional study

We first performed a cross-sectional study in which 105 healthy subjects (49 men and 56 women) aged 20–80 years participated. These subjects were divided into 2 groups. One group consisted of young (under 40 years, total: n = 45, age: 21.4 ± 0.5 years; males: n = 25, age: 22.0 ± 0.8 years; females: n = 20, age: 20.6 ± 0.2 years), while the second group consisted of middle-aged and older (over 40 years, total: n = 60, age: 67.4 ± 0.8 years; males: n = 24, age: 70.3 ± 1.2 years; females: n = 36, age: 65.4 ± 1.0 years) male and female subjects as outlined in Table 1. All subjects were recruited from a local community health center and a community recreation center. Subjects were excluded if diagnosed with hyperlipidemia, hypertension and hyperglycemia by a physician, taking anti-hyperlipidemic, anti-hypertensive, or anti-hyperglycemic medication, or had a history of stroke, diabetes, hypertension, hyperlipidemia, cardiac disease, chronic renal failure or mental disorders. None of the participants had a history of smoking for at least 12 months prior to the study. Women in the middle-aged and older group who had been postmenopausal for at least 5 years were not on hormone replacement therapy. All subjects were informed of the experimental procedures and risks, and provided written informed consent before participating in the study. The study was approved by the Ethics Committee of Ritsumeikan University and was conducted in accordance with the Declaration of Helsinki.

### Subjects: Interventional study

In the interventional study, 36 young (males: *n* = 20, age: 22.1 ± 1.0 years; females: *n* = 16, age: 20.4 ± 0.2 years) and 40 middle-aged and older (males: *n* = 16, age: 70.3 ± 1.5 years; females: *n* = 24, age: 64.8 ± 1.4 years) healthy subjects volunteered to participate. These subjects were sub-divided into two groups by including or not an aerobic exercise training intervention (AT). This subdivision resulted in the formation of four groups: a Young-AT group (*n* = 27), a Young control group (Young-Con, *n* = 9), a Middle-aged and older-AT group (*n* = 26), and a Middle-aged and older-Con group (*n* = 14) as depicted in Table 3. Subjects were recruited from the same local communities as in the cross-sectional study. Also, the same exclusion criteria were utilized. This study was registered at the University Hospital Medical Information Network Clinical Trials Registry (UMIN-CTR) (UMIN000035520).

### Experimental design

Measurements obtained for all subjects in the cross-sectional study included height, body weight, body mass index (BMI), resting heart rate (HR), resting systolic blood pressure (SBP), resting diastolic blood pressure (DBP), resting mean blood pressure (MBP), resting carotid-femoral pulse wave velocity (cfPWV), resting common carotid intima-media thickness (ccIMT), resting serum salusin-α levels and serum concentrations of total cholesterol, high-density lipoprotein (HDL) cholesterol and triglycerides. In the interventional study, 27 young (males: *n* = 15, age: 22.1 ± 1.0 years; females: *n* = 12, age: 20.4 ± 0.2 years) and 26 middle-aged and older (males: *n* = 10, age: 70.3 ± 1.5 years; females: *n* = 16, age: 64.8 ± 1.4 years) subjects completed an eight-week AT program. Measurements in this study were performed before and after AT and included height, body weight, peak oxygen uptake $\left({\dot{\text{V}}\text{O}}_{2\text{peak}}\right)$, resting SBP, resting DBP, resting cfPWV, resting HR, resting serum salusin-α levels and serum concentrations of total cholesterol, HDL cholesterol and triglycerides. At the beginning and end of the study period, fasting blood samples were drawn following at least 48 hours of rest after the last AT session to avoid the influence of acute effects of exercise. All subjects were instructed not to eat or drink fluids except for water for at least 12 hours before blood sampling. Serum samples were centrifuged (1500 x g, 15 min, 4°C) immediately after collection and stored at −80°C. All measurements were performed at a constant room temperature of 24°C.

### Exercise intervention

The subjects in the AT subgroups within the interventional study performed aerobic exercise training consisting of cycling on a leg ergometer (828E Monark cycle ergometer, Stockholm, Sweden) for 55 minutes, 3 days/week, for 8 weeks. Each 55-minute exercise bout consisted of a 5-minute warm-up period at 40% ${\dot{\text{V}}\text{O}}_{2\text{peak}},$ followed by 45 minutes of cycling at 60–70% ${\dot{\text{V}}\text{O}}_{2\text{peak}},$ and a 5-minute cool-down period at 40% ${\dot{\text{V}}\text{O}}_{2\text{peak}}.$ Exercise compliance was carefully monitored by direct supervision. We adopted this AT program in accordance with previous studies by our group demonstrating a favorable effect on arterial stiffness [9, 38]. The sedentary subjects in the control groups were instructed not to change their activities of daily living during the eight-week interventional period. Subjects in all groups were instructed to maintain their food intake as usual throughout the study.

### Measurement of VO_2peak_

${\dot{\text{V}}\text{O}}_{2\text{peak}}$ was measured during breath-by-breath oxygen consumption and carbon dioxide production using an incremental cycle exercise test on a cycle ergometer (MINATO, AE-310SRD, Osaka, Japan). To measure ${\dot{\text{V}}\text{O}}_{2\text{peak}},$ incremental cycle exercise began at a work rate of 60 W (30–90 W) for men and 30 W (0–60 W) for women, and power output was increased by 15 W/min until the subjects could not maintain a fixed pedaling rate of 60 rpm [9, 38]. Subjects were encouraged to exercise at maximum intensity throughout the ergometer test. Heart rate and rating of perceived exertion (RPE) were monitored every minute during the exercise bout. RPE was obtained using the modified Borg scale. The highest 30-second average value of ${\dot{\text{V}}\text{O}}_{2}$ during the exercise test was defined as ${\dot{\text{V}}\text{O}}_{2\text{peak}}$ if three out of four of the following criteria were met: (I) plateau in ${\dot{\text{V}}\text{O}}_{2}$ with an increase in external work, (II) maximal respiratory exchange ratio ≥ 1.1, (III) maximal heart rate ≥ 90% of the age-predicted maximum (208 − 0.7 × age; [39]), and (IV) RPE ≥ 18.

### Measurement of daily physical activity

Physical activity in freeliving conditions was evaluated with a triaxial accelerometer (Active style Pro HJA-350IT; Omron Healthcare, Kyoto, Japan) [9]. The accelerometer was placed at the waist (left side).

### Measurement of arterial stiffness, blood pressure, and heart rate

Subjects sat quietly for 30 minutes before measurements were acquired. Resting brachial artery SBP, DBP, MBP, cfPWV (an index of arterial stiffness) and HR were measured in duplicate in the supine position at rest using a vascular testing device (OMRON COLIN Co., Tokyo, Japan). Applanation tonometry was used to measure cfPWV using an array of 15 transducers, as previously described [9, 40]. PWV was calculated from the time delay between the carotid artery and femoral artery blood pressure waveforms and the distance between the two points, which was measured using a non-elastic tape measure. The coefficient of variation for interobserver reproducibility of cfPWV was 4.7% in this study. The mean value of SBP and DBP obtained at the left and right arms was calculated for analysis.

### Measurement of ccIMT

Images acquired with a Vivid S6 ultrasound system (GE Healthcare, Chicago, IL, USA) equipped with a high-resolution linear array transducer were used to measure ccIMT [41, 42]. Ultrasound images were analyzed by use of an image analysis software (Image J; National Institutes of Health, Bethesda, MD). At least 15 measurements of ccIMT were obtained for each segment and mean values were used for data analyses. The day-to-day coefficients of variation of this technique were 2.7 ± 0.7 %.

### Measurement of serum salusin-α levels

Serum salusin-α levels were measured in duplicate using an enzyme-linked immunosorbent assay (ELISA; Phoenix Pharmaceuticals, Burlingame, CA, USA). Optical density at 450 nm was measured using an xMark microplate reader (xMark microplate spectrophotometer; Bio-Rad Laboratories, Hercules, CA, USA). All samples were converted into concentrations by linear fitting to the log–log plot of a standard curve. The day-to-day coefficient of variation of serum salusin-α levels was 2.8 ± 0.7 %.

### Measurements of serum cholesterol and triglyceride levels

Fasting serum concentrations of total cholesterol, HDL cholesterol, and triglycerides were measured by standard enzymatic techniques.

### Statistical analysis

Data are expressed as means ± SE. In the cross-sectional study, unpaired Student’s t-tests were used to compare young versus middle-aged and older subjects for all parameters measured. Pearson correlation coefficients were used to assess the relationships between serum salusin-α levels with all other parameters obtained in this study. A multiple linear regression analysis was used to to test the independent association of serum salusin-α levels with BMI, triglyceride level, MBP, cfPWV. In the interventional study, unpaired Student’s t-tests were used to compare any parameter changes that occurred before and after the exercise intervention between the AT and control groups. As in the cross-sectional study, Pearson correlation coefficients were used to assess the relationships between serum salusin-α levels and the other parameters measured in all four groups of the interventional study. The partial correlation coefficient between serum salsuin-α levels and cfPWV, SBP or DBP was adjusted for age. A value of *P* < 0.05 was considered as statistically significant. All statistical analyses were performed using StatView (5.0, SAS Institute, Tokyo, Japan).
